# Predictors of health workers’ knowledge of maternal and perinatal deaths surveillance and response system in Morogoro region, Tanzania: An analytical cross-sectional study

**DOI:** 10.1097/MD.0000000000037764

**Published:** 2024-04-12

**Authors:** Christina Kashililika, Walter C. Millanzi, Fabiola Vincent Moshi

**Affiliations:** a Department of Clinical Nursing, School of Nursing and Public Health of the University of Dodoma, Dodoma, Tanzania; b Department of Nursing Management and Education, School of Nursing and Public Health of the University of Dodoma, Dodoma, Tanzania.

**Keywords:** health workers, knowledge, MPDSR system, predictors

## Abstract

This study aimed at assessing the predictors of knowledge about the Maternal and Perinatal Deaths Surveillance and Response (MPDSR) system among health workers in the Morogoro region. It was an analytical cross-sectional study, conducted from April 27 to May 29, 2020. A multistage sampling technique was used to recruit 360 health workers. A semi-structured questionnaire was used to collect the data. Statistical Package for Social Science (SPSS v.20) software was used for data entry and analysis. Bivariate and multivariate logistic regression analyses were used to assess factors associated with knowledge of MPDSR. A total of 105 (29.2%) health workers in the Morogoro region had adequate knowledge of the MPDSR system. After controlling for confounders, predictors of knowledge on the MPDSR system were the level of health facility a health worker was working (n [hospital [adjusted odds ratio [AOR] = 2.668 at 95% confidence intervals [CI] = 1.497–4.753, *P* = .001]), level of education of a health worker (diploma [AOR = 0.146 at 95% CI = 0.038–0.561, *P* = .005]), and status of training on MPDSR (trained [AOR = 7.253 at 95% CI = 3.862–13.621, *P* ≤ .001]). The proportion of health workers with adequate knowledge about the MPDSR system in the Morogoro region is unacceptably low. Factors associated with adequate knowledge were those working in hospitals with higher levels of professional training and those who had ever had training in MPDSR. A cost-effective strategy to improve the level of knowledge regarding MPDSR in this region is highly recommended.

## 1. Introduction

Health systems grounded in primary health care are vital to the health and well-being of women and newborns worldwide. Building a health workforce to meet the needs of women and newborns has become part of the world’s maternal and child health agenda through a sustainable development goal.^[1]^ Improving maternal and child health requires an increased commitment to and investment in the health workforce, which not only low- and middle-income countries struggle to meet needs and expectations but also promote and maintain good professional practices.^[2]^ Intense national, regional, and global efforts, supported by various partners/stakeholders, have led to significant progress in reducing maternal and newborn morbidity and mortality rates.^[3]^

Despite the increased essential maternal and newborn interventions worldwide, progress has not been universal as global estimates point to approximately 810 maternal deaths every day, 1 stillbirth every 16 seconds, and 2.4 million newborn deaths every year.^[4]^ Available statistics demonstrate that over 80% of under-five pediatric deaths and 86% of maternal deaths are concentrated in sub-Saharan Africa, where resources are constrained.^[5]^ Not surprisingly, the data may imply that maternal and newborn deaths in low- and middle-income countries are high at an estimated 415 maternal deaths/100,000 live births, with a lifetime risk as high as 1 in 37 among girls aged 15 years in Sub-Saharan Africa.^[6]^ The majority of reported maternal and newborn morbidity and mortality rates are more likely to be prevented with the development of competent, committed, and devoted health professionals who can provide evidence-based, cost-effective, and timely care to people.^[7]^

With the Coronavirus Disease of 2019 pandemic, some maternal and newborn healthcare interventions alongside services such as primary health care, including cervical cancer screening services, malaria-treated bed net distribution campaigns, immunization services, family planning, and antenatal care have been threatened.^[8]^ However, healthcare systems at the national, regional, and global levels struggle to establish systems and mechanisms to support network implementation of sexual and reproductive health interventions.^[9]^ Capacity building among healthcare workers has highlighted a significant contribution to the reduction in maternal and newborn morbidity and mortality rates by over 80%. The impact of national, regional, and global initiatives, such as the launching of maternal and perinatal death surveillance and response systems, have the potential to improve maternal and newborn health.^[10]^

The maternal and perinatal death surveillance and response system provides a roadmap for assuring the continuum quality of maternal and newborn health in clinical and policy settings.^[11]^ Although the implementation of the tool has been prescribed to focus primarily on district and health center levels, the process is linked to the surveillance, identification, notification, and review of community-based deaths.^[12]^ The aim is to make everyone in the health system responsible for reducing maternal and perinatal deaths. Moreover, the tool provides technical guidance for preventing maternal deaths and counting every baby born by auditing and reviewing stillbirths and neonatal deaths. For example, the auditing of maternal and perinatal deaths involves an effective in-depth investigation, identification, and reporting of the causes and circumstances surrounding them.^[13]^

Health workers oriented to maternal and perinatal death surveillance and response systems are believed to be able to identify health-related actions that may contribute to the timely prevention of further maternal and perinatal deaths.^[14]^ Nevertheless, health workers who are empowered adequately in the maternal and perinatal death surveillance and response system are expected to demonstrate competencies in assigning appropriate and timely health-related actions to particular groups or individuals, designating time frames for accomplishing the actions, and following up their implementations and effectiveness of pregnant mothers’ responses to care.^[15]^

Despite the adoption and implementation of the maternal and perinatal death surveillance and response system (MPDSRS) in different levels of health facilities to improve the well-being of pregnant women in Tanzania, the incidence and prevalence of maternal and perinatal deaths still prevail.^[16]^ This trend has been linked to unsatisfactory antenatal care attendance and the uptake of its associated services, as evidenced by the increased rate of home deliveries to approximately 60,000 in 2018, accounting for approximately 40% in the Morogoro region. Preventable obstetric complications during pregnancy, such as a ruptured uterus (1.9%), retained placenta (3.8%), sepsis (2.6%), obstructed labor (46.2%), pre/eclampsia (21.5%), and bleeding (23.9%), may be reduced with the effective and continuous implementation of the maternal and perinatal death surveillance and response system.^[17]^

Studies on the implementation of maternal and perinatal death surveillance and response systems for the prevention of maternal and perinatal deaths are becoming increasingly important for professionals and public health awareness.^[18]^ Given the persistent occurrence of maternal and perinatal deaths and/or home deliveries, there appears to be an unanswered question regarding whether health workers in the Morogoro region are aware of maternal and perinatal death surveillance and response systems. This study assessed predictors of health workers’ knowledge of the maternal and perinatal death surveillance and response system in the Morogoro region, Tanzania, with the aim of providing research-based information that may help launch educational interventions to empower them with competencies in the prevention of maternal and perinatal deaths in the region.

## 2. Methods

### 2.1. Study setting

This study was conducted in 3 districts of the Morogoro region: Morogoro Municipal, Mvomero District, and Kilosa District. The Morogoro region is 1 of the 31 regions in Tanzania. It has a population of 2,218,492 and consists of 7 districts and 9 councils (President’s Office, Regional Administration and Local Government, 2019). With 375 health facilities, the Morogoro region is among the top 5 regions in Tanzania with a high number of health facilities. The total number of skilled healthcare workers employed by the government was 2846. The Morogoro region was chosen as the location of the study because of its high maternal and perinatal mortality rates and the fact that no study related to MPDSR had been conducted in the region before.

### 2.2. Study design

A hospital-based analytical cross-sectional study employing a quantitative approach was used. The study population comprised of health workers who were responsible for participating in the MPDSR system.

### 2.3. Study population

The study included health workers who participated in the MPDSR meetings. The inclusion criteria for this study were all healthcare workers who were in service for at least 1 year before the data collection period, and the exclusion criteria were all health workers who were working in the health facility on a part-time basis.

### 2.4. Sample size estimation and sampling technique

#### 2.4.1. Sample size estimation.

Several participating health facilities (n_1_) and health workers (n_2_) were calculated from the formula for a cross-sectional study of a finite population (Dahiru, Aliyu, & Kene, 2006), as shown in Equation 1.^[19]^

$$\begin{array}{l} n=\frac{\frac{t^{2}P(1P)}{e^{2}}}{1+\frac{1}{N}\left(\frac{\;t^{2}P\left(1P\right)1}{e^{2}}\right)} \end{array}$$ (Equation 1)

where, *N* = the size of the target population, t = the critical value for the given confidence level, in this study the confidence level was 95%, therefore t was equal to Z_0.05_ which is 1.96, e = the margin error, taken for 0.05.

The proportion of health workers who had adequate knowledge of the MPDSRS system from previous studies was 60.6% (Agaro et al, 2016).

From the above formula, the sample size for health workers “n_1_” was given as follows:


$$\begin{array}{l} n_{1}=\frac{\frac{{\left(1.96\right)}^{2}\times 0.606(10.606)}{{\left(0.05\right)}^{2}}}{1+\frac{1}{2846}\left(\frac{\;{\left(1.96\right)}^{2}\times \;0.606\left(10.606\right)\;1}{{\left(0.05\right)}^{2}}\right)} \end{array}$$

$$\begin{array}{l} n_{1}=360 \end{array}$$

The minimum required sample size was 360 healthcare workers.

#### 2.4.2. Sampling technique.

A multistage sampling technique was applied for the facility selection. Three councils from the Morogoro region were selected based on the large number of health facilities. The facilities selected were Morogoro Municipal Council, Mvomero District Council, and Kilosa District Council. Because the 3 councils had varying numbers of health facilities, the stratification method was used to allocate 10 facilities to the Morogoro Municipal Council, 15 to the Mvomero District Council, and 13 to the Kilosa District Council. On each council, hospitals were purposively selected because of their relatively higher volumes of deliveries and the fact that hospitals manage most of the complications of pregnancy and childbirth, while health centers were randomly selected.

A sample of 360 health workers was equally distributed across the 3 councils, with each council having 120 health workers. Each council facility contributed an equal number of health workers: 12 from each facility in the Morogoro Municipal Council, 8 from each facility in the Mvomero District Council, and 9 or 10 from each facility in the Kilosa District Council.

Finally, the selection of health workers from each facility was done randomly by the lottery replacement method from a list of health workers in the attendance register on the day of data collection that was obtained from the office of the facility in charge.

### 2.5. Data collection technique and tool

A semi-structured questionnaire developed using the MPDSRS system guide was used to collect data on knowledge of the MPDSRS system among health workers in the Morogoro Region.

### 2.6. Variable measurements.

Knowledge of MPDSR among health workers was defined in this study as the extent to which health workers have a theoretical and practical understanding of the MPDSR system. Knowledge of MPDSR among healthcare workers was measured through a questionnaire having 20 statements with binary responses; “True” and “False” where a participant was asked to select either of the 2 responses by putting a tick in a respective box. A correct response was awarded one (1) point, while an incorrect response was a zero (0) points. A total score of 15 points or above was considered “adequate knowledge” and a score of 14 points or below was considered “inadequate knowledge.” Overall knowledge of MPDSRS was a categorical variable, with one (1) given to those with adequate knowledge and zero (0) for those with inadequate knowledge.

### 2.7. Data analysis procedure

The obtained quantitative data were analyzed using SPSS software for both descriptive statistics (frequency distribution and Chi-square) and inferential statistics (binary logistic regression analysis) to predict factors associated with knowledge of the MPDSR system among health workers in Morogoro. The statistical limit was set at a 95% confidence interval and 5% significance level. The findings are presented in figures and tables as frequencies (n) and percentages (%).

## 3. Results

### 3.1. Socio-demographic characteristics

The findings in Table 1 show that 360 health workers from hospitals and health centers participated in the study, with a response rate of 100%. The study findings revealed that 69.7% (n = 251) of the respondents were aged 30 years or above, and 69.8% (n = 244) were females. About 52.5% (n = 189) of the respondents had an education diploma level, while those with the lowest and highest education levels were certificate holders 40.6% (n = 146) and bachelor holders 2.5% (n = 9), respectively. In contrast, nurses accounted for 54.2% of the sample (n = 195). The percentage of respondents who were trained on the MPDSRS was 21.4% (n = 77) (Table 1).

**Table 1:** Demographic characteristics of the study respondents (N = 360).

Variables	Frequency (%)
Age group	
<30 years	109 (30.3)
≥30 years	251 (69.7)
Sex	
Male	116 (32.2)
Female	244 (67.8)
Education	
Certificate	146 (40.6)
Diploma	189 (52.5)
Advanced diploma	16 (4.4)
Bachelor	9 (2.5)
Profession	
Nursing	195 (54.2)
Clinician	63 (17.5)
Others	102 (27.3)
Duty station	
Antenatal ward	66 (18.3)
Labor ward	101 (28.1)
Postnatal ward	69 (19.2)
OPD	70 (19.4)
Others	54 (15.0)
Duration in practice	
1–5 years	136 (37.8)
6–10 years	128 (35.6)
Above 10 years	96 (26.6)
Training on the MPDSR system	
Yes	77 (21.4)
No	283 (78.6)

### 3.2. Knowledge of health workers on the MPDSR system

**Figure 1.**
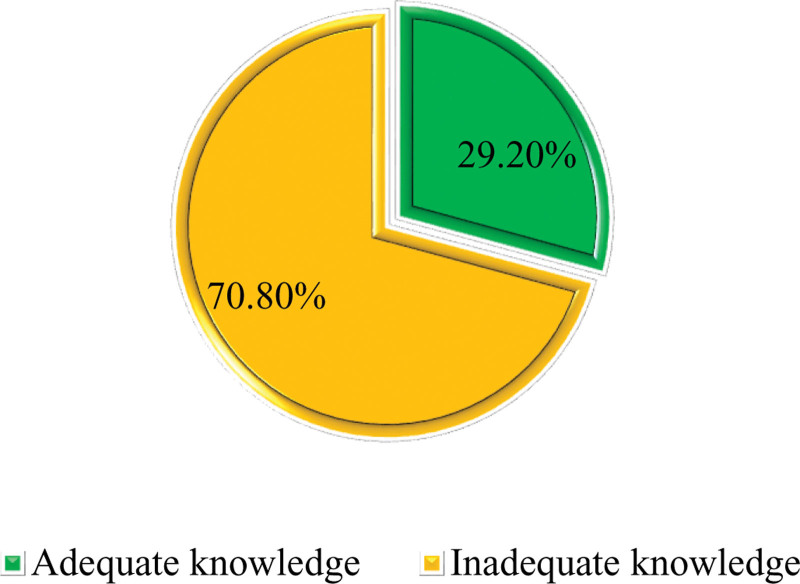
Proportional distribution of knowledge about the MPDSR system among study participants.

The findings in Figure 1 show that the average score among health workers was 13.52 points with maximum and minimum scores of 20 and 7 out of 20, respectively. The findings in Figure 1 indicate that 29.2% (n = 105) of the study respondents had adequate knowledge of the MPDSR program, while 70.8% (n = 255) had inadequate knowledge about it (Fig. 1).

#### 3.2.1. Item distribution of knowledge of health workers on MPDSRS.

Table 2 shows the findings of the item analysis used to measure knowledge and the distribution of scores for each item. The findings indicated that 73.6% of the study respondents were not knowledgeable that the hospital nurse in charge was responsible for preparing the clinical summary for every maternal and perinatal death occurring in the respective health facility. Moreover, 71.4% of them were not knowledgeable that the identification, notification, and review of community-based maternal and perinatal deaths are not part of the MPDSR, but they may be linked in the process. Nevertheless, 68.1% of them did not know that, depending on the circumstances of death, sometimes the atmosphere of “NO shame NO blame NO name” could not be maintained during the review meetings.

**Table 2:** Item distribution of knowledge of health workers on the MPSDR system.

Item	Correct n(%)	Incorrect n(%)
Deaths must be notified within 24 hours of occurrence	351 (97.5)	9 (2.5)
Maternal death review includes investigations of the circumstances surrounding the death.	310 (86.1)	50 (13.9)
The “Response” part of MPDSR deals with the implementation of action plans from maternal/perinatal death reviews.	322 (89.4)	38 (10.6)
Near misses are also part of MPDSR review meetings	143 (39.7)	217 (60.3)
Identification, notification, and review of community-based maternal and perinatal deaths are not part of MPDSR	103 (28.6)	257 (71.4)
In Tanzania maternal and perinatal deaths are notifiable events; thus, it is an offense if not notified.	301 (83.6)	59 (16.4)
The facility focal person is not allowed to use a cell phone to notify deaths.	256 (71.1)	104 (28.9)
All facilities should have daily zero reporting of maternal/perinatal deaths	127 (35.3)	233 (64.7)
Every facility is required to have an MPDSR committee	323 (89.7)	37 (10.)
Collectively, the members of the MPDSR committee need to have the expertise to identify the medical (not necessarily nonmedical) problems that contributed to the deaths.	292 (81.1)	68 (18.9)
The MPDSR focal person of the hospital or health center is the secretary of an MPDSR committee.	306 (85.0)	54 (15.0)
Facility committees should be involved in the review of community maternal and perinatal deaths	167 (46.4)	193 (53.9)
The hospital nurse in charge is responsible for preparing the clinical summary for every death.	95 (26.4)	265 (73.6)
The deaths review should be done within 7 days of occurrence for facility deaths and at most 14 days for community deaths.	295 (81.9)	65 (18.5)
Strengths observed during care should be identified and acknowledged in review meetings.	324 (90.0)	36 (10.0)
Clinical summaries of deaths are prepared by the MPDSR focal person BEFORE the review of each maternal/perinatal death.	287 (79.7)	73 (20.3)
Clinical notes are not required during review meetings but the MPDSR committee may request the clinical case notes during the discussions if they are deemed to be necessary.	120 (33.3)	240 (66.7)
The clinical summary should include information from an interview with health workers who took care of the deceased	317 (88.1)	43 (11.9)
Depending on the circumstances of the deaths, sometimes the atmosphere of “NO shame NO blame NO name” cannot be maintained during the review meetings	115 (31.9)	245 (68.1)
Action plans during the MPDSR meeting must be SMART	3147.2)	46 (12.8)

Some of the study respondents (66.7%) and (64.7%) did not know that clinical notes are not required during review meetings, but the MPDSR committee may request the clinical case notes during the discussions if they are deemed to be necessary and if all facilities should have daily zero reporting of maternal/perinatal deaths, respectively, 60.3. Of the respondents, 60.3% did not know that near misses were also part of MPDSR review meetings for the prevention of maternal and perinatal deaths in their health facilities.

### 3.3. The relationship between health workers’ characteristics and knowledge of MPDSRS

As shown in Table 3, the variables that showed a significant relationship with knowledge of MPDSR were health facility level (X^2^ = 11.97, *P* = .01), age group of respondents (X^2^ = 12.12, *P* = .005), gender of respondents (X^2^ = 5.933, *P* = .015), level of education (X^2^ = 31.04, *P* < .001), profession (X^2^ = 80.59, *P* = .016), duty station (X^2^ = 13.20, *P* = .01), and status of training on MPDSR (X^2^ = 48.16, *P* < .001). See Table 3.

**Table 3:** The relationship between health workers’ characteristics and knowledge of the MPDSR system.

Variable	Inadequate n(%)	Adequate n(%)	X^2^	*P*-value
Characteristics of health facility				
Hospital	68 (37.4)	114 (62.6)		
Health center	37 (20.8)	141 (79.2)	11.97	.001
Location of health facility				
Urban	39 (32.2)	82 (67.2)		
Rural	69 (27.6)	173 (72.4)	0.829	.393
Age group				
Below 30	18 (16.5)	91 (83.5)		
30 and above	87 (34.7)	164 (65.3)	12.12	.005
Sex				
Male	24 (20.7)	92 (79.3)		
Female	81 (33.2)	163 (66.8)	5.953	.015
Level of education				
Certificate	44 (30.1)	102 (69.9)		
Diploma	42 (22.2)	147 (77.8)		
Advanced diploma	12 (75.0)	4 (25.0)		
Bachelor	7 (77.8)	2 (22.2)	31.04	<.001
Profession				
Nurse	67 (34.4)	128 (65.6)		
Clinician	19 (30.2)	44 (69.8)		
Others	19 (18.6)	83 (81.4)	8.059	.016
Duty station				
Antenatal ward	20 (30.3)	46 (69.7)		
Labor ward	39 (38.6)	62 (61.4)		
Postnatal ward	23 (33.3)	46 (66.7)		
OPD	16 (22.9)	54 (77.1)		
Other units	7 (13.0)	47 (87.0)	13.20	.01
Training on MPDSRS				
Yes	47 (61.0)	30 (39.0)		
No	58 (20.5)	225 (79.5)	48.16	<.001

### 3.4. The association between health workers’ characteristics and knowledge of MPDSRS

After controlling for confounders, Table 4 indicates the variables that showed significant association with the knowledge of health workers in the MPDSR program, which included the level of a health facility [Hospital (AOR = 2.668 at 95% confidence intervals [CI] = 1.497–4.753, *P* = .001), level of education (Diploma [AOR = 0.146 at 95% CI = 0.038–0.561, *P* = .005)], and status of training on MPDSR [Yes (AOR = 7.253 at 95% CI = 3.862–13.621, p = <0.001)], as shown in Table 4.

**Table 4:** Association between health workers’ characteristics and knowledge of MPDSRS.

Variable	OR	95%CI		*P*-value	AOR	95%CI		*P*-value
		Lower	Upper			Lower	Upper	
Level of facility								
Health center	1				1			
Hospital	2.273	1.420	3.638	.001	2.668	1.497	4.753	.001
Age group								
<30 yrs.	1				1			
≥30 yrs.	0.373	0.211	0.658	.001	1.579	0.307	1.092	.091
Sex								
Male	1				1			
Female	0.525	0.311	0.885	.016	0.650	0.351	1.204	.171
Education								
Bachelor	1				1			
Adv. diploma	1.510	0.923	2.471	.101	1.139	0.615	2.111	.679
Diploma	0.144	0.144	0.471	.001	0.146	0.038	0.561	.005
Certificate	0.123	0.123	0.617	.011	0.209	0.035	1.234	.084
Profession								
Nurse	1				1			
Clinician	1.212	0.656	2.240	.539	0.568	0.215	1.502	.254
Others	2.287	1.281	4.082	.005	2.272	0.763	6.765	.140
Duty station								
Antenatal ward	1				2			
Labor ward	0.691	0.357	1.338	.273	0.945	0.432	2.064	.887
Postnatal ward	0.870	0.421	1.796	.706	0.431	0.154	1.204	.108
OPD	1.467	0.682	3.157	.327	2.490	0.838	7.398	.101
Other units	2.919	1.127	7.563	.027	1.120	0.259	4.840	.879
Training								
No	1				1			
Yes	6.078	3.537	10.44	.001	7.253	3.862	13.62	.001

## 4. Discussion

The findings of this study reveal that the majority of health workers do not know the concept of MPDSRS, its characteristics, or its implementation in the prevention of maternal and perinatal deaths in their working stations. Although the MPDSRS is used in health facilities to identify, analyze, report, and prevent the aforementioned deaths, the majority of health workers were not knowledgeable that the hospital nurse in charge was responsible for preparing the clinical summary for every maternal and perinatal death occurring in the respective health facility. Moreover, they were not knowledgeable that the identification, notification, and review of community-based maternal and perinatal deaths are not part of the process, but they may be linked in the process. Nevertheless, others did not know that, depending on the circumstances of death, sometimes the atmosphere of “NO shame NO blame NO name” could not be maintained during the review meetings.

Despite being the health team front-liners in implementing the MPDSRS, other health workers did not know that clinical notes were not required during review meetings, but the MPDSRS committee may request the clinical case notes during the discussions if they are deemed to be necessary and that all facilities should have daily zero reporting of maternal/perinatal deaths. Moreover, some health workers did not know that near misses were also part of MPDSRS review meetings for the prevention of maternal and perinatal deaths in their health facilities. Health workers’ knowledge of the MPDSRS observed in this study was significantly associated with their sociodemographic characteristics, including working in hospitals, having diploma education, and having a history of being trained on the MPDSRS.

This study observed that working in hospitals is a positive precursor to health workers’ knowledge of the MPDSRS. Based on the Tanzanian healthcare system, health workers in hospitals are privileged because many of them are strategically well equipped with not only medical supplies and equipment but also continuum opportunities for professional support, mentorships, on-job training, seminars, inter-professional meetings/discussions and or workshops and conferences than other low levels do. Needless to say, being exposed to previous training about MPDSRS would probably enhance health workers’ knowledge about it, as they would remember and refer to the content they were exposed to.

Health workers with diploma education being knowledgeable about the MPDSRS may be argued in this study because their proportional distribution was higher than that of other higher levels of education. However, having a diploma education was significantly associated with knowing MPDSRS than having a certificate education because the more an individual advances in their education career, the more they become oriented and aware of the respective phenomena. Many studies have previously demonstrated that despite the adoption and implementation of the MPDSRS, the majority of health workers do not know well about it. Among the few studies, Ayele et al,^[11]^ highlighted that some of the challenges to the effective implementation of maternal and perinatal death surveillance and response systems include sociodemographic characteristics profiles of health workers and institutional factors.

Basu et al,^[20]^ revealed the knowledge of health workers at the MPDSRS that the majority of them did not demonstrate good knowledge before being trained about it, and their characteristics, training package, and institutional factors influenced their gain in knowledge after the training. Moreover, the study by Namagembe et al,^[21]^ revealed that one of the barriers to the effective implementation of MPDSRS was the inadequate knowledge of health workers about it and fear of blame. Most of the barriers to its implementation were found to be more health worker-based than others.

In Tanzania, the study conducted by Mutabazi et al,^[16]^ in Mtwara revealed that of all other factors, health workers’ knowledge about the MPDSRS recommendations contributed to its ineffective implementation and, thus, was linked to the occurrence of maternal and perinatal deaths. In addition, Tayebwa et al,^[22]^ reported that the MPDSRS is not implemented as recommended because, among others, health workers lack competencies about it, and as a result, maternal and perinatal death tolls. The findings of the current study are similar to those of previous studies, probably because all studied the implementation of MPDSRS and health workers’ competencies on it. It has been observed that health workers still have no adequate knowledge about the MPDSRS; thus, training may help empower them to know and implement it as recommended, and more effectively and efficiently. The findings of this study were posted in a research square and are available online^[23]^

## 5. Limitation

The study was not without limitations, as a quantitative study could have limited the description of the predictors of knowledge. In addition, by its nature, it lacked a causal effect relationship; this limitation was minimized by including many variables that were controlled during data analysis.

## 6. Conclusion

The proportion of health workers with adequate knowledge about the MPDSR system in the Morogoro Region is unacceptably low. Factors associated with adequate knowledge were those working in hospitals with higher levels of professional training and those who had ever had training in MPDSR. A cost-effective strategy to improve the level of knowledge regarding MPDSR in this region is highly recommended.

## Acknowledgments

The authors thank the University of Dodoma for providing ethical clearance for this study. We are also grateful to the administration of the Morogoro Region for allowing us to conduct the study, and to each of the study participants for their participation in this study.

## Author contributions

**Conceptualization:** Christina Kashililika, Fabiola Vincent Moshi.

**Data curation:** Christina Kashililika.

**Formal analysis:** Christina Kashililika.

**Methodology:** Christina Kashililika.

**Resources:** Christina Kashililika.

**Supervision:** Walter C. Millanzi, Fabiola Vincent Moshi.

**Writing – original draft:** Walter C. Millanzi, Fabiola Vincent Moshi.

**Writing – review & editing:** Walter C. Millanzi, Fabiola Vincent Moshi.
